# Operative versus conservative treatment of acute Achilles tendon ruptures: preliminary results of clinical outcome, kinematic MRI and contrast-enhanced ultrasound

**DOI:** 10.1007/s00402-022-04457-7

**Published:** 2022-05-14

**Authors:** Juana Kosiol, Alexander Keiler, Alexander Loizides, Hannes Gruber, Benjamin Henninger, Andreas Bölderl, Leonhard Gruber

**Affiliations:** 1 Department of Orthopaedics and Traumatology, Medical University of Innsbruck, Anichstrasse 35, 6020 Innsbruck, Austria; 2 Department of Radiology, Medical University of Innsbruck, Anichstrasse 35, 6020 Innsbruck, Austria

**Keywords:** Achilles tendon, Acute Achilles tendon rupture, Kinematic MRI, Contrast-enhanced ultrasound, Operative treatment, Conservative treatment, Clinical outcome

## Abstract

**Introduction:**

There is no uniform consensus on the gold standard therapy for acute Achilles tendon rupture. The aim of this pilot study was to compare operative and conservative treatment regarding imaging findings and clinical outcome.

**Materials and methods:**

Surgically or conservatively treated patients with acute Achilles tendon rupture were retrospectively evaluated. Differences in tendon length and diameter with and without load were analysed using kinematic MRI, tendon perfusion, structural alterations, movement and scar tissue by means of grey-scale and contrast-enhanced ultrasound (CEUS). Intra- and interobserver agreement were recorded.

**Results:**

No significant difference was detected regarding clinical outcome, B mode ultrasonography, contrast-enhanced sonography or MRI findings, although alterations in MRI-based measurements of tendon elasticity were found for both groups. Considerable elongation and thickening of the injured tendon were detected in both groups.

**Conclusion:**

Both, conservative and surgical treatment showed comparable outcomes in our preliminary results and may suggest non-inferiority of a conservative approach.

**Supplementary Information:**

The online version contains supplementary material available at 10.1007/s00402-022-04457-7.

## Introduction

There is still no consensus on whether acute Achilles tendon ruptures should be treated conservatively or operatively [1]. Supporters of conservative treatment argue that there is no risk for surgery-related complications such as wound healing problems, scar tissue, adhesion of the tendon, infection or nerve damage [2]. According to the current literature, two major indications for surgical treatment are: tendon gap formation of more than 1 cm in 20° plantar flexion or treatment initiation more than 24 h after injury, where haematoma formation is likely. In the latter case, elongation of the tendon is quite likely and might lead to a functionally unsatisfactory outcome in conservative treatment. The main reason for supporting operative treatment is based on lower rerupture rates [3]. The aftercare protocols also differ considerably and range from early functional rehabilitation models to strict immobilisation for six to eight weeks. However, a current meta-analysis showed that early functional rehabilitation protocols reduce the rerupture rate in non-operatively treated patients to a level similar to that of patients treated by surgery [4, 5].

Contrast-enhanced ultrasound (CEUS) can be used to detect neovessels, angiogenesis and perfusion with greater sensitivity than duplex ultrasound (DU) following Achilles tendon rupture [6]. The advantage of kinematic magnetic resonance imaging (MRI) is that it enables the visualisation of pathologies that depend on the position and/or load in the area of interest. This technique was performed mainly to detect pathologies related to joints regarding the musculoskeletal domain [7]. Only a few studies have been published using kinematic MRI to clarify the involvement of tendons such as peroneal tendon subluxation or biceps tendon dislocation [8, 9]. To our knowledge, no other study has used kinematic MRI or CEUS to compare the outcome of operative and non-operative treatment after Achilles tendon rupture.

The aim of this study was to compare conservatively and operatively treated acute Achilles tendon ruptures regarding (1) clinical examination (2) ultrasound parameters and (3) MRI findings. Ethics approval was obtained from the local Ethics Committee (No. 2016-0010).

## Materials and methods

The study design was retrospective comparative. Patients treated for acute Achilles tendon rupture between January 2010 and June 2017 were identified retrospectively and reviewed according to our inclusion and exclusion criteria (Table 1). Overall, 63 patients could be identified. 52 patients (4 females and 48 males) of these met our inclusion criteria. All were contacted by phone and invited to participate in the study. 18 patients, all male, with a median age of 52.2 years agreed and were included in the study. Nine of them had had non-operative treatment and nine operative treatment. All of the surgically treated patients had undergone open Achilles tendon repair with a double-bound Bunnell technique (also described as bundle-to-bundle suture technique) [10, 11], followed by a strict functional rehabilitation protocol with full weightbearing in boot with an equinus of 20° plantar flexion immediately after surgery. Patients were immobilised in boot for six weeks and planter flexion was reduced every two weeks, ending in a neutral position after four weeks. All of the conservatively treated patients had also undergone a strict functional rehabilitation protocol with immediate full weightbearing in boot, however at full equinus with 30° plantar flexed. Patients were immobilised in boot for eight weeks and planter flexion was reduced every two weeks, ending in a neutral position after six weeks. After the period of immobilisation, patients in both groups started formal physical therapy in a full active range of motion. The median time between treatment onset and study follow-up was 26 months. All patients signed the informed consent form after receiving oral and written information. To compare the structural and functional outcome after operative or non-operative treatment of acute Achilles tendon rupture, prospective imaging methods and a clinical examination were performed.

**Table 1:** Inclusion and exclusion criteria

Inclusion criteria	Exclusion criteria
• Age ≥ 18 • Signed consent document • Availability for follow-up • Living within greater town area • No contraindication for contrast-enhanced ultrasound • No contraindication for MRI • Operative/conservative treatment of an acute Achilles tendon rupture at least six months before examination • No previous Achilles tendon rupture on the same or opposite side	• Age < 18 • Skin problems/chronic wound in the lower extremity • Previous fracture/surgery on the lower leg in the last six months • Open Achilles tendon ruptures • Treatment onset later than one week after the accident • Previous Achilles tendon ruptures on the opposite side • Arthrodesis of the ankle joint • Rheumatoid arthritis of the ankle joint • Neurological disease • Pregnancy/ breastfeeding • Allergy to ultrasound contrast agent

Clinical examination included the following parameters: first, the circumference of both, the upper and the lower leg (10 cm above/below the patella midline) was measured on both, the injured and the healthy side. Second, the range of motion of the upper ankle joint (talocrural joint) on both sides was measured with a goniometer (active dorsiflexion and plantar flexion). In addition, each patient was requested to walk normally, to stand on the foot/tiptoes/heels with both legs simultaneously and with each leg separately. It was recorded whether the patient was confident when performing the exercise, whether he was able to perform the exercise and whether differences between the injured and the non-injured side were found in performance, height or extent of standing on the foot/tiptoes/heels. The Achilles Tendon Total Rupture Score (ATRS) [12, 13] was calculated as well. This score contained ten questions for grading limitations, difficulties and pain following Achilles tendon rupture from daily routine to sports activity. The highest possible score was 100 points. The full clinical examination was conducted each time by the same blinded senior consultant, who was not involved in surgical treatment of any patient.

A functional, a duplex and a contrast-enhanced ultrasound were performed by an experienced radiologist using an Epiq 7 ultrasound device (Philips Healthcare, Amsterdam, Netherlands) with a linear transducer 3–12 MHz Functional Ultrasound, providing a real-time image of the tendon’s gliding and the characteristics of the scar. For visualisation of perfusion by means of neovessels [14] (up to 40 µm), 4.8 ml of the contrast agent SonoVue (Bracco International, Milan, Italy) and 10 ml NaCl 0.9% were applied via a 21 gauge indwelling cannula placed in the brachial vein. Flow was detected over a three-minutes examination interval with a pre-set low mechanical index (MI) between 0.05 and 0.07. The mechanical index expressed an approximation to the maximum amplitude of the pressure pulse in the tissue. It was determined with the ultrasound’s peak negative pressure (PNP) and the centre frequency (Fc); MI = PNP/√(Fc).

**Fig. 1:**
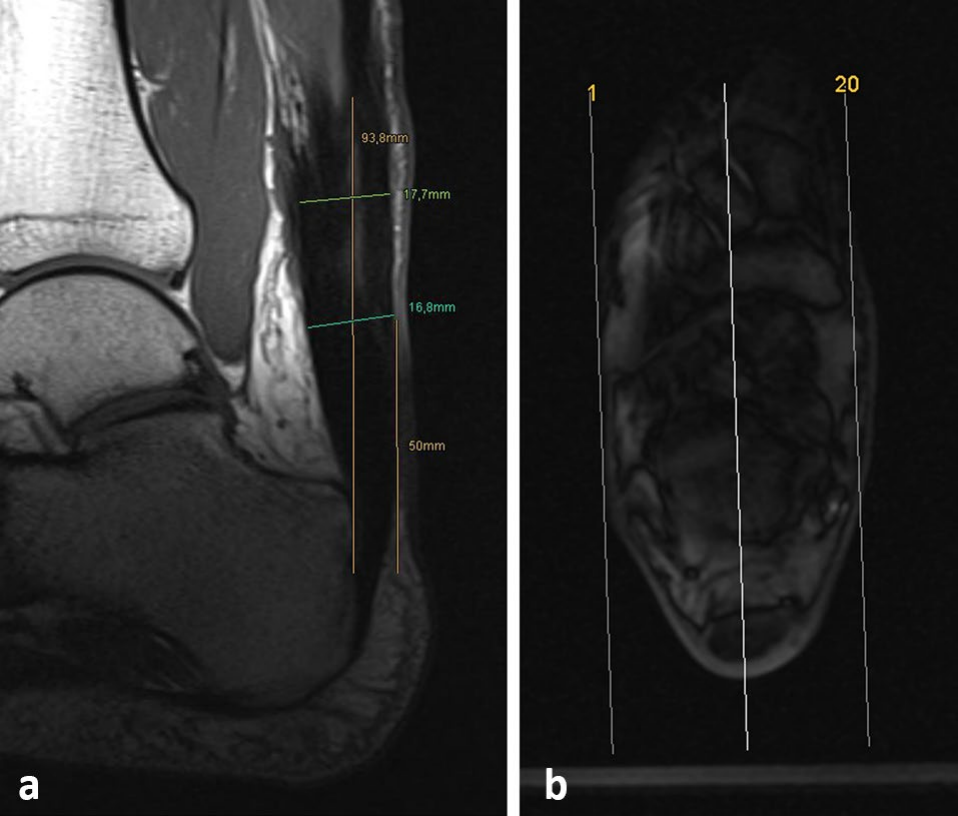
MRI measuring technique. Measuring the length of the tendon from the insertion to the musculotendinous junction, the diameter of the tendon at its most bulky point and 5 cm above the insertion (**a**) using the localizer in the second layer to determine the tendon midline (**b**)

To obtain information about the tendon’s elasticity by means of changes in the length and diameter of the tendon, an MRI (without and with a load of 3 bars) in neutral and loaded position was performed of the injured and contralateral Achilles tendon in 17 patients using a Magnetom Skyra 3 Tesla MRI (Siemens AG, Berlin, Germany). For the kinematic examination, a CE-accredited ergometer (Trispect, Ergospect GmbH, Innsbruck, Austria) was used. The magnetic resonance-compatible diagnostic pedal was intended for defined stressing of the thigh musculature to support a diagnostic examination in a 3 Tesla whole-body MRI. It contained the following components: a diagnostic pedal with fastening straps for the lower leg, a central control unit including the controller and the interface, optical light-wave conductors with Ethernet connection and USB voltage supply for connecting the work station to the controller, optical light-wave conductors for connecting the controller to the interface, sensor cable to connect the interface to the diagnostic pedal, air pressure hose for supplying the controller with compressed air from an external compressed air system and an air pressure hose for supplying the examination module with compressed air from the controller. After connecting all components, the ergometer was operated and controlled with a computer using Trispect software (Ergospect GmbH, Innsbruck, Austria). Patients were asked to plantar flex the ankle joint against the pedal, where a resistance of 3 bars was applied, and to hold this position for two-minutes intervals. The length of the tendon from its insertion at the calcaneus to the musculotendinous junction, the calibre 5 cm proximal of the tendon insertion and the largest diameter were measured in the tendon midline (Fig. 1) in the neutral position images and in the loaded images. For comparison, the healthy side of each patient was abducted.

### Statistics

MRI scans were evaluated by an orthopaedic surgeon and a radiologist according to the intra- and interobserver principle (two evaluations were made by both investigators with a time lag of at least one week).

All data were collected and stored in Microsoft Excel (Microsoft Corporation, Redmond, Washington, USA). Statistical analysis was carried out in GraphPad Prism 8.1.1 (GraphPad Software Inc., La Jolla, California, USA) and SPSS Statistics 23.0 (IBM Corporation, Armonk, New York, USA).

The 95% confidence intervals (CI) were given in nonparametric variables and as mean ± standard deviation (SD) in those with a normal distribution. *P* values < 0.05 were considered statistically significant, and adjustment for multiple testing was performed where appropriate. Continuous data are presented as box plots including median and lower/upper quartiles; whiskers denote 5% and 95% percentiles. Outliers are presented as circles. Contingency data are presented as columns denoting percentage of total (frequency).

Interobserver agreement was calculated via intraclass correlation coefficients (based on a two-way random effect model). Results are provided as intraclass correlation coefficients with 95% confidence intervals. Data distribution of continuous variables was assessed with a D’Agostino & Pearson normality test. An unpaired T test (with Welch correction, if necessary) or Mann–Whitney test were used to compare variables if no correction for multiple testing was necessary. An ordinary one-way ANOVA was used to compare measured values with a Holm-Sidak correction in the case of multiple testing.

Contingency tables were analysed via Fisher’s exact test or a χ^2^ test, depending on the table dimensions (2 × 2 or greater).

## Results

### Patient population

**Fig. 2:**
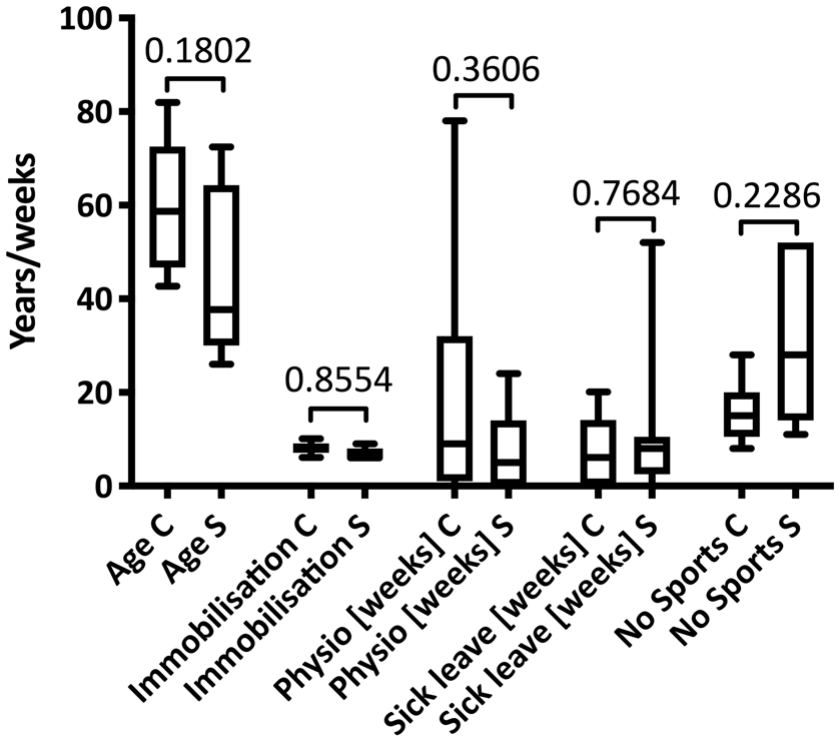
Demographics. Comparison of age, immobilisation duration, physiotherapy duration, sick leave duration and sports abstinence in participants having undergone conservative (C) or surgical treatment (S) after Achilles tendon rupture

Table 2 gives an overview of participant demographics. There were no significant differences between the conservative and the surgical group regarding duration of immobilisation (*p* = 0.855), duration of physiotherapy (*p* = 0.360), duration of sick leave (*p* = 0.768), duration of physical inactivity (i.e., no sports) (*p* = 0.228) or age (*p* = 0.180) (Fig. 2, Table 2).

**Table 2:** Participant demographics

	Overall	Conservative treatment	Surgical treatment	*p* value
*n*	18	9	9	–
Age [years]	52.2 ± 16.8	59.2 ± 14.1	45.2 ± 18.0	0.180^§^
Male sex [%, *n*]	100.0% (18)	100.0% (9)	100.0% (9)	> 0.999^$^
Left side [%, *n*]	66.6 (12)	55.5 (5)	77.7 (7)	0.620^$^
Follow-up interval [months]	25.9 ± 18.3	17.4 ± 10.9	34.5 ± 21.7	0.093^§^
Immobilisation duration [weeks]	7.4 ± 1.4	8.0 ± 1.4	6.8 ± 1.2	0.855^§^
Physiotherapy duration [weeks]	13.9 ± 20.9	18.6 ± 27.4	7,9 ± 8.6	0.360^§^
Sick leave [weeks]	9.1 ± 11.9	6. 9 ± 7.6	11.2 ± 15.8	0.768^§^

^§^Unpaired *T* test (Welch correction applied, if necessary) or Mann–Whitney test

^$^χ^2^ test

### Clinical examination

**Fig. 3:**
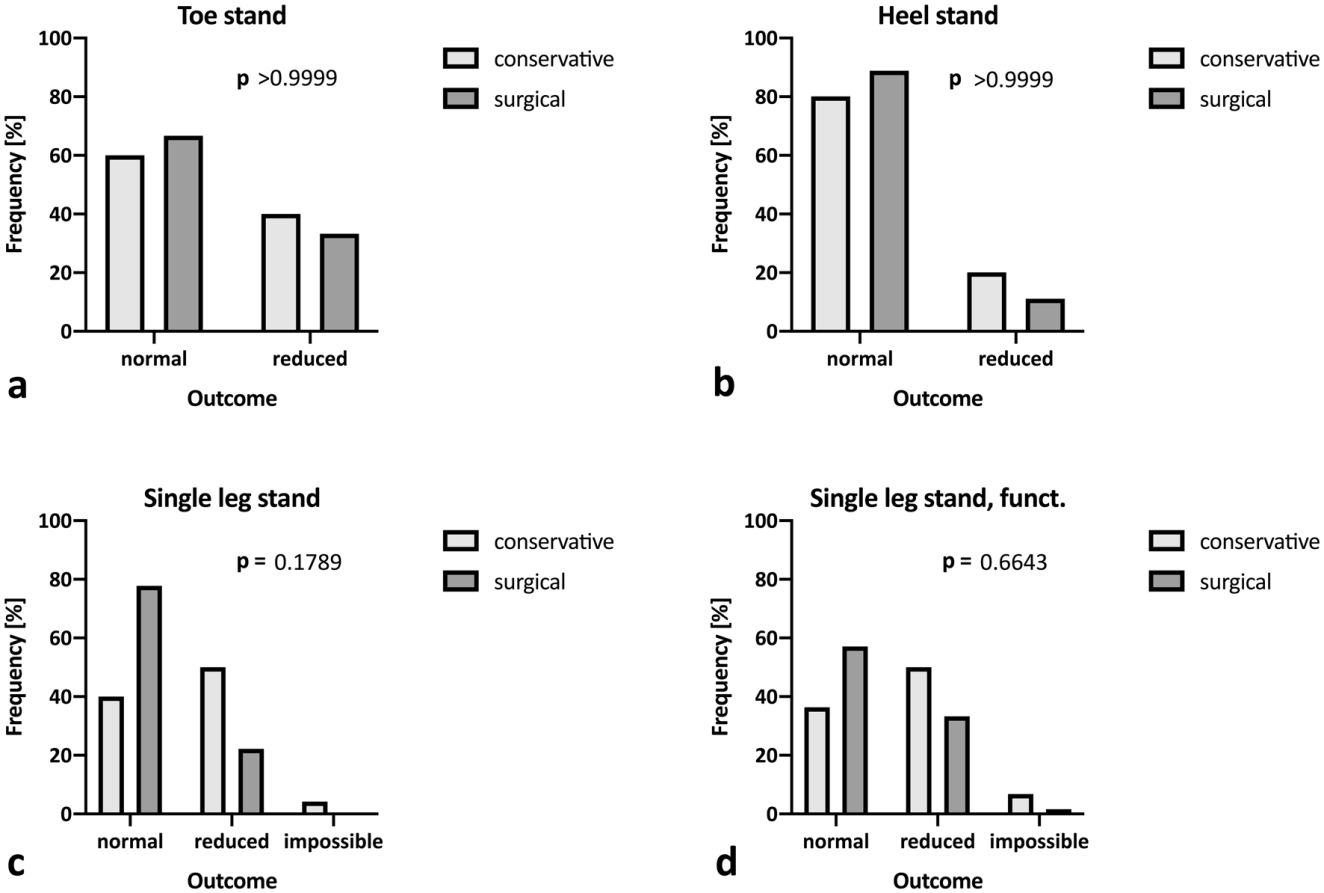
Clinical examination. Comparison of clinical findings after conservative (light grey) and surgical treatment (dark grey) after Achilles tendon rupture

**Fig. 4:**
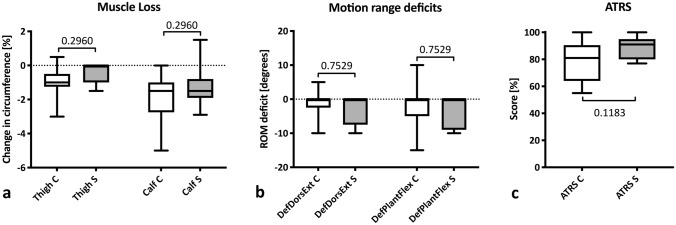
Functional outcome. Changes in thigh and calf circumference (Fig. 4a) and motion range deficits (Fig. 4b) compared to the healthy side as well as the result of Achilles Tendon Total Rupture Score (ATRS) (Fig. 4c) in participants having undergone conservative (C) or surgical treatment (S) after Achilles tendon rupture

Each patient underwent a clinical examination. No significant differences were found between the injured and the non-injured side in performance or height of toe stand (*p* = 0.999; Fig. 3a) or heel stand (*p* = 0.999; Fig. 3b). Although higher rates of unstable single-leg stand (*p* = 0.179; Fig. 3c) and unstable dynamic single-leg stand (*p* = 0.664; Fig. 3d) were observed in the conservative group, differences did not reach the significance level (Table 3). There was no significant difference in the amount of muscle loss (*p* = 0.296) for either thigh or calf; Fig. 4a), motion range deficits for both dorsal extension and plantar flexion (*p* = 0.753; Fig. 4b) or Achilles tendon rupture scores (*p* = 0.118; Fig. 4c, Table 3). No rerupture was encountered in either group during the observation period.

**Table 3:** Overall, conservative and surgical treatment group results

		Overall	Conservative group	Surgical group	*p* value^$^
Clinical examination	Impaired toe stand [%/*n*]	50 (9)	44.4 (4)	33.3 (3)	0.999
	Impaired heel stand [%/*n*]	16.7 (3)	22.2 (2)	11.1 (1)	0.999
	Unstable static single leg stand (reduced/impossible) [%/*n*]	44.4 (8)	44.4 (4)/22.2 (2)	22.2 (2)/0.0 (0)	0.179
	Unstable dynamic single leg stand (reduced/impossible) [%/*n*]	33.3 (6)	22.2 (2)/0.0 (0)	33.3 (3)/11.1 (1)	0.664
	Muscle loss (thigh circumference) [cm]	− 0.7 ± 0.8	− 1.0 ± 1.0	− 0.4 ± 0.6	0.296
	Muscle loss (calf circumference) [cm]	− 1.5 ± 1.3	− 1.9 ± 1.5	− 1.2 ± 1.2	0.296
	Motion range deficit: plantar flexion [°]	− 2.9 ± 4.3	− 1.7 ± 7.1	− 3. 1 ± 4.7	0.753
	Motion range deficit: dorsal extension [°]	− 1.4 ± 5.4	− 1.1 ± 4.2	− 2.8 ± 4.4	0.753
	Achilles Tendon Total Rupture Score	83.4 ± 12.4	78.7 ± 14.9	88.2 ± 8.3	0.118
Ultrasonographic examination	Visible force transmission [%/*n*]	58.8 (10)	55.5 (5)	62.5 (5)	0.664
	Tendon adhesion [%/*n*]	58.8 (10)	55.6 (5)	62.5 (5)	0.664
	Bridging scar [%/*n*]	94.1 (16)	100.0 (9)	87.5 (7)	0.999
	Tendon mobility [%/*n*]	76.5 (13)	77.8 (7)	75.0 (6)	0.999
	Tendon vascularisation [%/*n*]	5.9 (1)	11.1 (1)	0.0 (0)	0.999
	Contrast-enhancement (yes/no) [%/*n*]	88.2 (15)	100.0 (9)	75.0 (6)	0.559
	CE Δtime-to-peak (fat vs. tendon) [sec]	2.3 ± 11.8	6.9 ± 9.2	− 6.8 ± 8.0	0.021
	Tendon CE time-to-peak [sec]	19.7 ± 12.9	21.05 ± 14.23	18.15 ± 15.32	0.622
	Fat CE time-to-peak [sec]	17.3 ± 8.5	20.9 ± 7.5	20.9 ± 9.2	0.440
	CE AUC [dB*sec]	64.8 ± 68.2	60.39 ± 54.50	69.76 ± 88.51	0.999
MRI measurements	Tendon length, rest (injured) [mm]	116.1 ± 20.4	118.1 ± 20.41	113.9 ± 21.43	0.912
	Tendon width, rest (injured) [mm]	11.5 ± 3.2	10.73 ± 3.926	12.16 ± 2.480	0.996
	Relative rest tendon length (compared to healthy side) [FOB]	1.4 ± 0.3	1.5 ± 0.3	1.4 ± 0.3	> 0.999
	Relative rest tendon width (compared to healthy side) [FOB]	2.2 ± 1.1	2.1 ± 0.9	2.4 ± 1.3	0.968
	Relative tendon elongation under stress [FOB]	1.5 ± 0.3	1.5 ± 0.3	1.5 ± 0.3	> 0.999
	Relative tendon width under stress [FOB]	2.1 ± 0.9	2.1 ± 0.8	2.1 ± 1.1	> 0.999

Overview of functional, sonographic and MR tomographic findings

*CE* contrast enhancement, *AUC* area under the curve, *FOB* fold over basal

^$^Comparison between conservative and surgical group

### Achilles tendon ultrasonographic examination

**Fig. 5:**
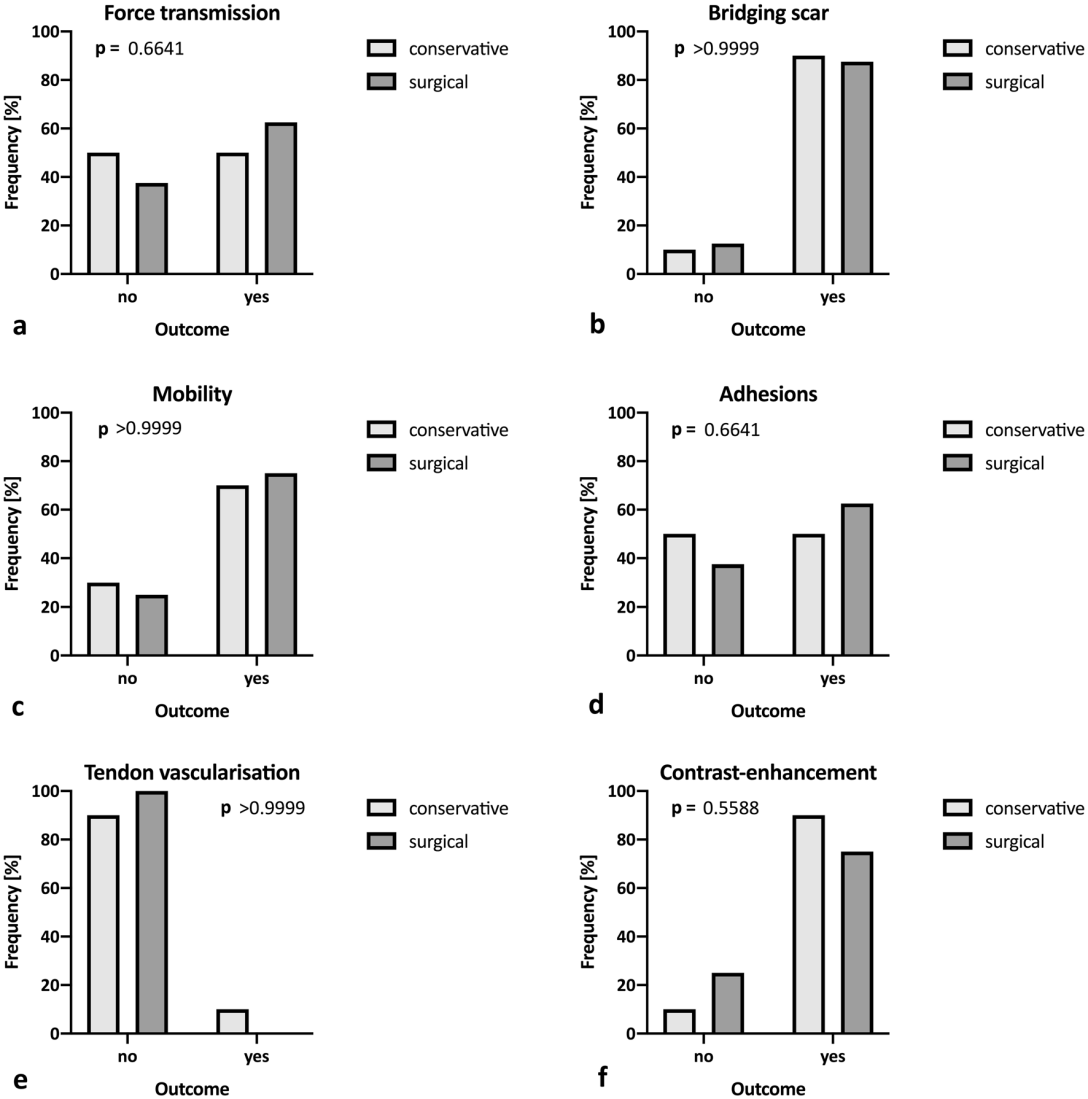
Ultrasonographic examination. Comparison of dynamic B mode findings for force transmission (**a**), bridging scar (**b**), tendon mobility (**c**) and presence of adhesions (**d**) as well as intratendinous Doppler vascularisation (**e**) and presence of contrast enhancement (**f**)

No difference in visible force transmission (*p* = 0.664; Fig. 5a) or in the rate of adhesions (*p* = 0.664; Fig. 5d) was observed on dynamic B mode sonography following surgical treatment. No significant differences were found with regard to the rate of bridging scar formation (*p* = 0.999; Fig. 5b), tendon mobility (*p* = 0.999; Fig. 5c) or tendon vascularisation visualised by Doppler mode (*p* > 0.999; Fig. 5e). One ultrasound dropout occurred in the operative group due to lost follow-up (patient was not willing to undergo ultrasound investigation). Furthermore, no significant difference was found with regard to tendon contrast enhancement (*p* = 0.559; Fig. 5f, Table 3).

**Fig. 6:**
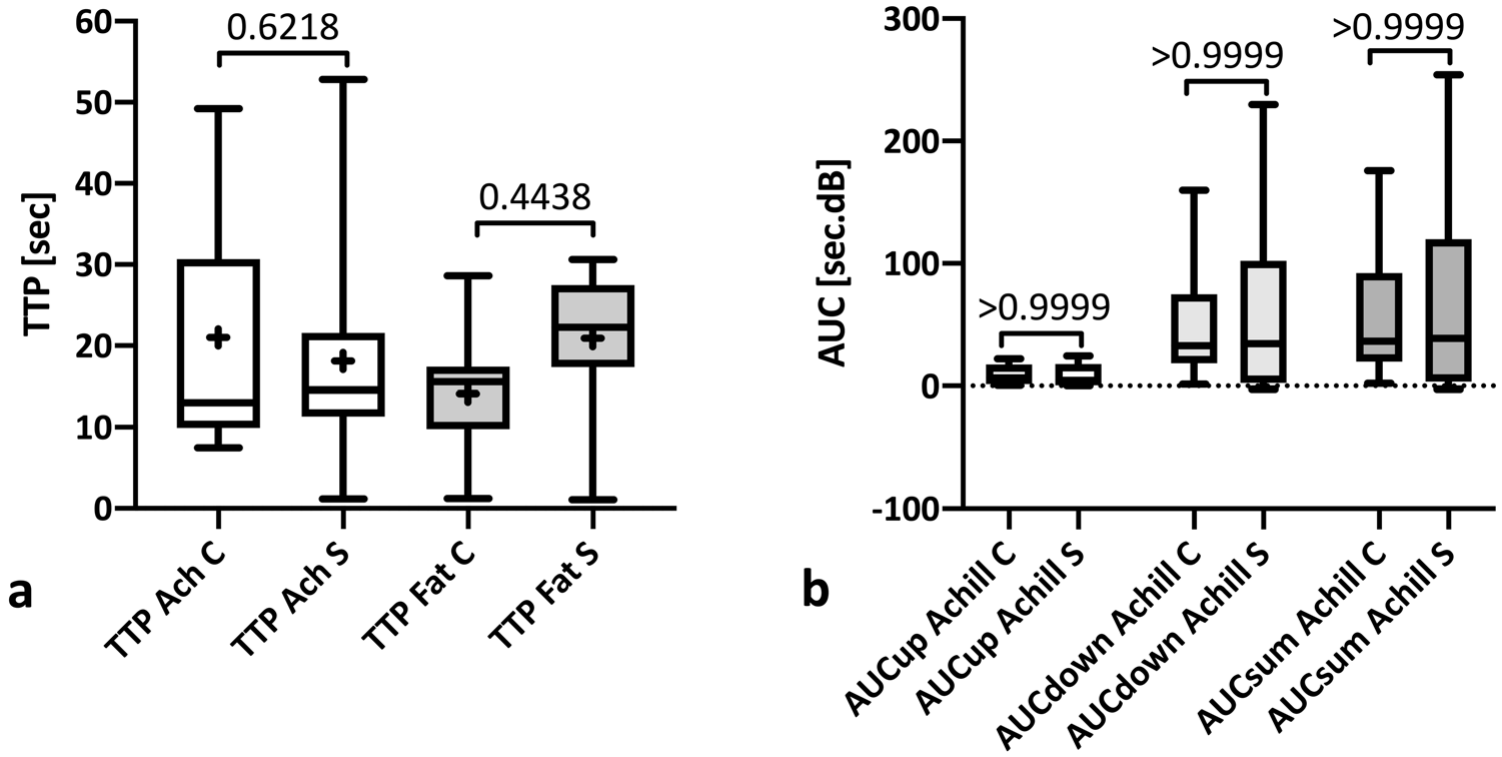
Contrast-enhanced ultrasound. Comparison of contrast-enhanced ultrasound (CEUS) parameters time-to-peak (TTP) for injured Achilles tendons, surrounding fat and resulting TTP differences (**a**) and CEUS area-under-the-curves (AUC) for inflow (AUCup), outflow (AUCdown) and total observation period (AUCsum) in participants having undergone conservative (C) or surgical treatment (S) after Achilles tendon rupture (**b**)

Dynamic contrast-enhancement analysis revealed a significantly lower ΔTTP (time-to-peak) after surgical treatment (6.9 ± 9.2 s vs. − 6.8 ± 8.0 s; *p* = 0.021), which resulted from a non-significantly greater postsurgical peritendinous fat tissue contrast enhancement (TTP 20.9 ± 7.5 s) than for conservative treatment (TTP 20.9 ± 9.2 s; *p* = 0.44; Fig. 6a, Table 3).

No differences were found regarding the total contrast enhancement flow detected in the area under the curve between the conservative and the operative group (*p* = 0.999; Fig. 6b, Table 3).

### Achilles tendon MRI measurements

**Fig. 7:**
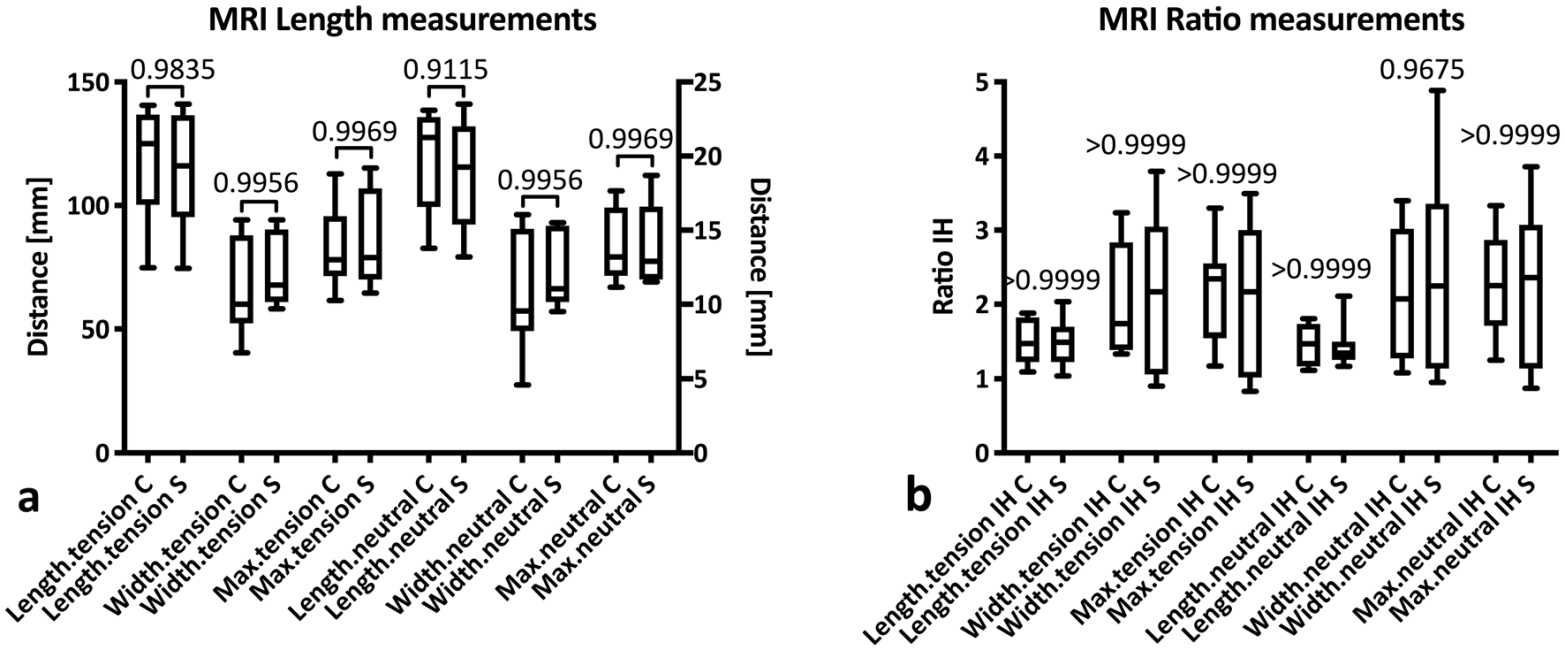
Conservative versus surgical treatment in MRI. Comparison of MRI tendon length and diameter measurements in participants having undergone conservative (C) or surgical (S) treatment (**a**) and comparison of MRI tendon measurement ratios between injured (I) and healthy (H) sides in participants having undergone conservative (C) or surgical (S) treatment after Achilles tendon rupture (**b**)

Inter-reader agreement was ‘high’ for all MRI-based measurements. No significant differences with regard to tendon dimensions or length at rest or under tension were observed between the conservative and the surgical group (Fig. 7a, Table 3) and no significant differences were found when comparing the injured (I) and the healthy (H) side in the conservative vs. the surgical group (Fig. 7b, Table 3). One dropout from MRI measurement occurred in the non-operative group (patient was not willing to undergo MRI investigation).

### Comparison of injured and healthy tendons across the whole study population

**Fig. 8:**
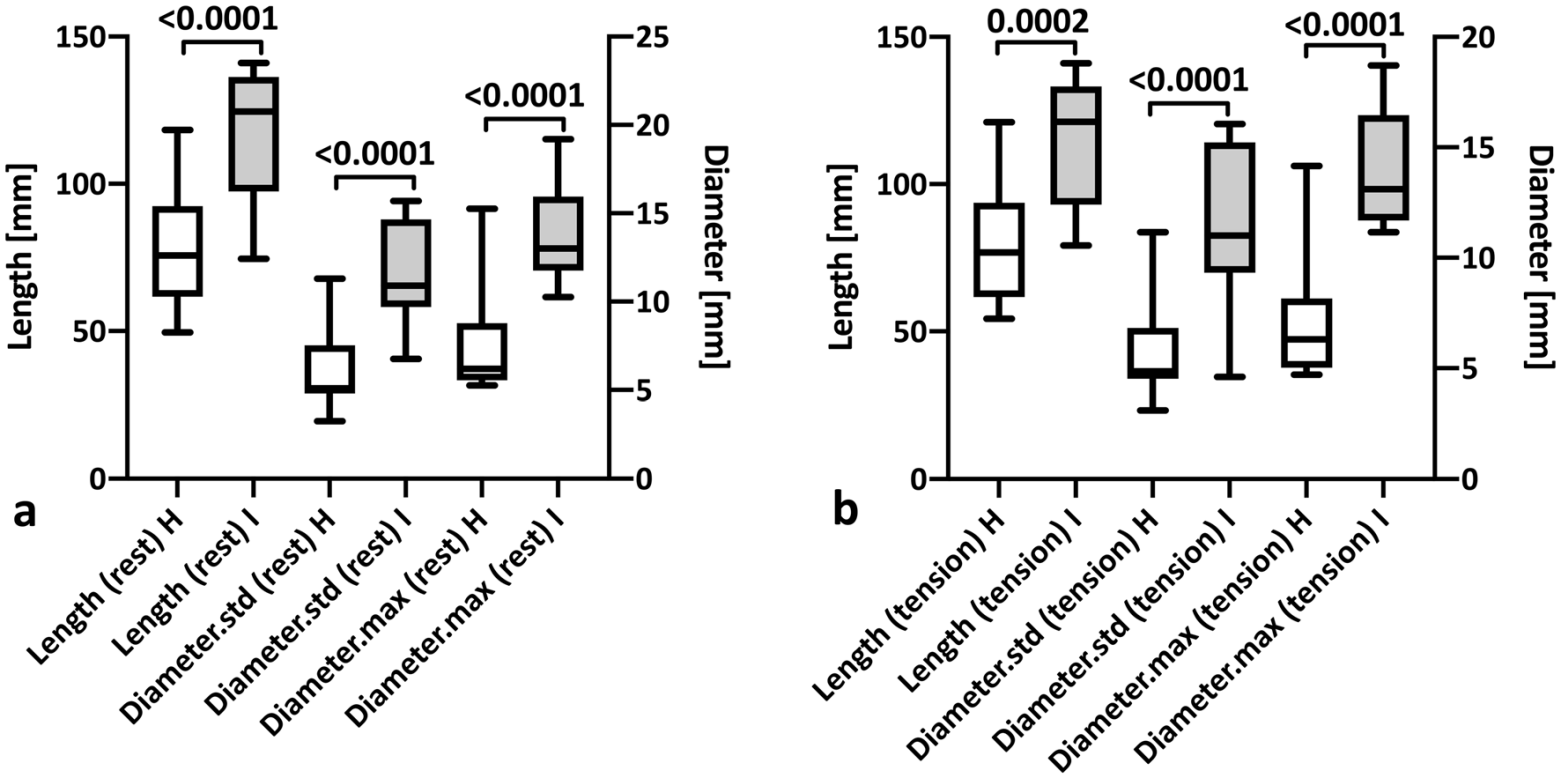
Healthy versus injured tendons in MRI. Difference in Achilles tendon length and diameter at rest (**a**) and under tension (**b**) when comparing the injured (I) and the healthy (H) side

Standardised MRI measurements showed a highly significant elongation and thickening of injured tendons at rest (Fig. 8a, Table 3) and under tension (Fig. 8b, Table 3). Injured Achilles tendon length increased by 50.9% compared to the healthy tendon (*p* < 0.0001), tendon diameter by 113.0% (*p* < 0.0001) for standardised measurements and maximum diameter by 111.3% (*p* < 0.0001; Fig. 8a). The same results were found for tendons under tension (+ 44.4%, 124.5% and 125.6%, respectively; Fig. 8b, Table 3).

## Discussion

The most important finding was that, in general and regarding all parameters, no statistically significant difference was detected when comparing the conservative versus the surgical group. However, comparison of all injured tendons and all healthy and non-injured tendons showed considerable elongation and thickening of the injured tendon, regardless of whether treatment was conservative or operative. When summarizing all measurements made in the conservative and the operative group and comparing them with the measurements made in the non-injured side, the length of the Achilles tendon on the injured side was seen to be increased by 50.9% at rest and by 44.4% under load. The tendon diameter on the injured side was increased to 113.0% at rest and to 124.5% under load, with no statistically significant difference seen for operative or conservative treatment. Moreover, the healthy tendon showed a statistically insignificant tendency to shorten from neutral to plantar flexed (loaded) position of -3.5 mm on average. In contrast, the injured tendon showed no change in length between those two positions, leading to the assumption that the tendon loses elasticity after a rupture due to intratendinous repair tissue.

To our knowledge, no other study has used kinematic MRI to evaluate structural alteration and performance of the Achilles tendon under load to compare results following operative and non-operative treatment. With this advantage, we observed changes in the length and diameter of the tendon between rest and under push-off simulation when comparing the injured and the healthy tendon as well as the surgically and the conservatively treated tendon. The considerable elongation measured by us could have been induced not only by scar formation in the tendon, but also by the reduced volume and fascicle length of the gastrocnemius and soleus muscle complex. This might give the tendon a relatively longer appearance when measuring its length from its insertion in the calcaneus to the musculotendinous junction.

Heikkinen et al. presented results related to the presented study [15]. Their prospective study investigated the long-term follow-up after surgical repair of Achilles tendon rupture in 56 patients, correlating MRI findings and clinical examination. Achilles tendon length, calf muscle volume, and muscle fatty degeneration were assessed, comparing the injured and the healthy side. They describe an average lengthening of the tendon of 12 mm accompanied by 11%–13% smaller calf muscle volumes and persistent plantar flexion strength deficits after surgical repair of Achilles tendon rupture, even 13 years after the injury. Nicholson et al. evaluated morphological changes in the muscle–tendon unit after surgical repair of an Achilles tendon rupture, taking the healthy tendon as comparison [16]. In the ultrasound measurements, the muscle fascicle length of the gastrocnemius and soleus muscle complex was shortened by 12.1%-19.6%, muscle thickness was reduced by 9.1%–20.1% and the tendon cross-sectional area showed an elongation of 46.7% + -34.5%. In contrast to the current study, no data on conservative treatment were collected in either of the two studies mentioned above.

According to the current literature, the main benefit of surgical treatment can be found in a reduced rerupture rate [17]. On the other hand, surgical treatment of Achilles tendon rupture leads to an increased risk for complications such as deep vein thrombosis, wound healing problems and scar adhesions [18–21]. Yang et al. and Gwynne-Jones et al. pointed out that the rerupture rate can be influenced positively by early functional rehabilitation protocols [22, 23], as tendons need a functional stimulus for healing and parallel orientation of collagen fibres [24, 25]. Manent et al. also presented comparable results in a prospective, randomised study comparing conservative and operative treatment using the same early weight-bearing and functional rehabilitation protocol for all groups [26]. Thirty-four patients were included and randomised to conservative treatment, open surgery or percutaneous surgery. Clinical outcome parameters, muscle strength and calf measurements as well as the ATRS, the Victorian Institute of Sport Assessment (VISA) Questionnaire and an ultrasound examination after one year that assessed tendon length, diameter and perfusion were evaluated. Although the results showed no statistically significant difference among the groups, problems with wound healing and adhesion were detected in the groups after operative treatment. The dynamic B mode sonography examination in the current study supports the findings made in the study by Manent et al. as we observed a non-significant higher rate of adhesions on dynamic B mode sonography after surgical treatment as well as a non-significant, slightly higher rate of visible force transmission. Lantto et al. compared surgery and conservative treatment in a randomised, controlled study evaluating clinical outcome and calf muscle strength recovery [27]. The authors observed superior performance of surgery in restoring calf muscle strength of the triceps surae muscle compared to the conservative treatment. The outcome scores showed similar results in both groups. However, neither operative nor conservative treatment restored the muscle to the same strength as on the healthy side. Bruns et al. support this finding. In an animal in vivo model with sheep, the Achilles tendon was transected 3 months, 6 months and 12 months after spontaneous healing and evaluated for histology and mechanical strength. The histological structure of the healed tendon was comparable to that of the healthy side, but showed clearly decreased strength and decreased total rupture force in biomechanical testing [28]. In a recent retrospective cohort study, Wenning et al. observed persisting functional deficits at more than 3 years following Achilles tendon repair ranging from strength deficits to specific impairments of functional performance [29]. In a prospective randomized clinical trial of open operative, minimally invasive and conservative treatments of acute Achilles tendon tear, no significant difference was found in the functional outcome when patients were treated operatively or conservatively at a 24 month follow-up. Sonographically, all patients showed isolated structure loosening and a significantly thickened cross-sectional area compared with the non-injured opposite side, without differences between the groups [5]. These results correlate well with our findings where, regardless of the selected treatment, the injured tendon remained elongated, thickened and inferior in tissue quality. Accordingly, in our evaluation of all clinical outcome parameters, no statistically significant differences were detected.

Interestingly, the dynamic contrast-enhancement analysis revealed a significantly shorter time-to-peak after surgery, based on non-significantly higher postsurgical peritendinous fat tissue contrast-enhancement as compared to conservative treatment. This finding could be related to changes in skin and fat tissue due to the surgical approach after Achilles tendon rupture repair. Contrast-enhanced ultrasound (CEUS) can be used to detect neovessels, angiogenesis and perfusion with a higher sensitivity than duplex ultrasound (DU) after Achilles tendon rupture [6]. On the basis of microbubbles, CEUS enables vessels as small as 40 µm to be visualised, compared to 100 µm with DU [30, 31]. The most important role of this imaging method is in cancer diagnosis and aftercare [31, 32]. Fischer et al. demonstrated in a pilot study that CEUS is a feasible method for assessing microperfusion in a reconstructed tendon [33].

As in our evaluation, the area under the curve for inflow, outflow and the total observation period showed in summary the same contrast enhancement flow for the conservative and the operative treatment group, suggesting that there is no irritation or chronic inflammation of the suture fibre remaining inside the tendon after surgical repair.

Limitations of our pilot study are certainly the quite small patient cohort and the lack of randomisation of the selected treatment. Inherently, no data on patients not fulfilling the inclusion criteria, such as young patients without any possible age-related degenerative damages of the Achilles tendon, could be gathered. This might lead as well to a kind of exclusion bias. The fact that we could not find any statistically significant difference between the operative and the non-operative treatment could also be caused by a beta error. In addition, patients were selected retrospectively, which is why the level of evidence in this study is low.

Another limitation is the lack of documentation of possible comorbidities of our patients, due to the characteristics of a feasibility study. Though, there might be therefore a bias to unhealthier or “unathletic” patients in the conservative group. On the other hand, the decision to treat patients operatively or non-operatively is based on radiological findings (extent of dehiscence of the ruptured tendon), choice of the treating physician and on the patient’s preference. Therefore, there is a possibility of bias towards surgery in physically active and/or younger individuals. However, as we present a strictly retrospective analysis with a representative patient collective, potential bias is also reflective of current clinical practice at our hospital. Further studies should elucidate whether functional status influences surgical decision making and introduces an a priori bias.

## Conclusion

Our preliminary results show that with regard to the observed outcome parameters, conservative and surgical treatment are equivalent treatment options for acute Achilles tendon rupture. Regardless of the selected treatment, the injured tendon remains elongated, thickened and inferior in tissue quality, leading to less elasticity and strength compared to the healthy tendon. A prospective, randomised study with a larger number of patients is necessary to clarify the tendency to better force transmission and more adhesions of the tendon after operative treatment as well as the implied differences in contrast enhancement between the two groups.

## Supplementary Information

Below is the link to the electronic supplementary material.

Supplementary file1 (JPG 149 KB)
