# Supplementary figures and images for: Operative versus conservative treatment of acute Achilles tendon ruptures: preliminary results of clinical outcome, kinematic MRI and contrast-enhanced ultrasound
Source: Arch Orthop Trauma Surg. 2022 May 14;143(5):2455–65. doi: 10.1007/s00402-022-04457-7 (PMC10110640; doi:10.1007/s00402-022-04457-7)

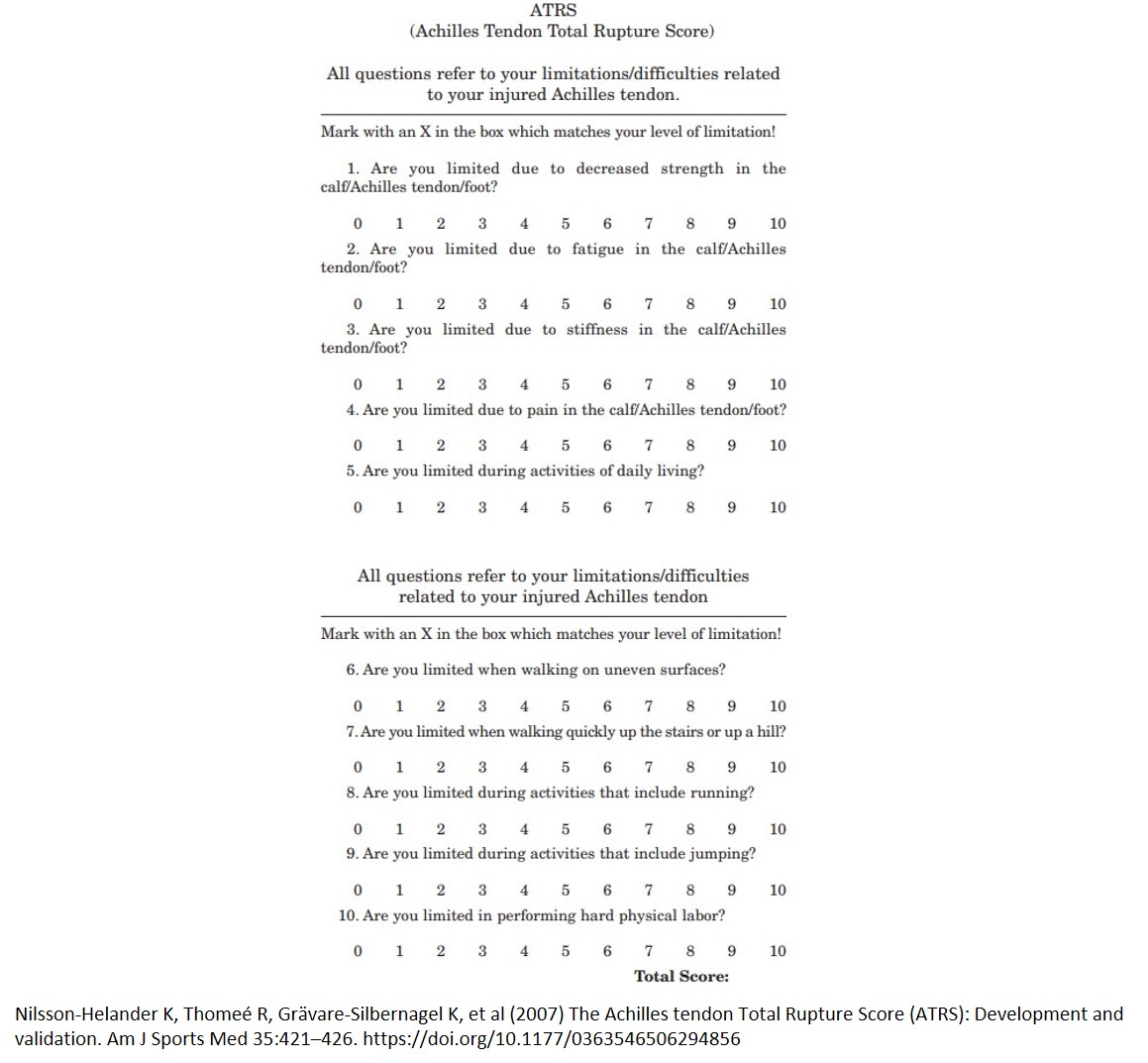

Supplement: Supplementary file 1 — Supplementary file1 (JPG 149 KB) [file 402_2022_4457_MOESM1_ESM.jpg]
